# Feeding ecology in sea spiders (Arthropoda: Pycnogonida): what do we know?

**DOI:** 10.1186/s12983-018-0250-4

**Published:** 2018-03-15

**Authors:** Lars Dietz, Jana S. Dömel, Florian Leese, Tobias Lehmann, Roland R. Melzer

**Affiliations:** 1 Zoological Research Museum Alexander Koenig, Statistical Phylogenetics and Phylogenomics, Adenauerallee 160, D-53113 Bonn, Germany; 2 Faculty of Mathematics and Natural Sciences, University of Bonn, D-53012 Bonn, Germany; 3 Aquatic Ecosystem Research, Faculty of Biology, University Duisburg-Essen, Universitätsstr. 5, D-45141 Essen, Germany; 4 Bavarian State Collection of Zoology – SNSB, Münchhausenstraße 21, 81247 Munich, Germany; 5 Department Biologie II, Ludwig-Maximilians-Universität München, Großhaderner Straße 2, 82152 Planegg-Martinsried, Germany; 6 GeoBioCenter LMU, Richard -Wagner-Str. 10, 80333 Munich, Germany

**Keywords:** Pantopoda, Marine arthropods, Food chain, Benthos, Community ecology

## Abstract

Sea spiders (Pycnogonida) are a widespread and phylogenetically important group of marine arthropods. However, their biology remains understudied, and detailed information about their feeding ecology is difficult to find. Observations on pycnogonid feeding are scattered in the literature, often in older sources written in various languages, and have never been comprehensively summarized. Here we provide an overview of all information on feeding in pycnogonids that we have been able to find and review what is known on feeding specializations and preferences in the various pycnogonid taxa. We deduce general findings where possible and outline future steps necessary to gain a better understanding of the feeding ecology of one of the world’s most bizarre animal taxa.

**Electronic supplementary material:**

The online version of this article (10.1186/s12983-018-0250-4) contains supplementary material, which is available to authorized users.

## Background

Sea spiders (Pycnogonida) are a phylogenetically distinct group of marine arthropods with about 1500 species. General reviews of their biology were provided by King [1] and Arnaud & Bamber [2]. Almost all species have a holobenthic lifestyle. They are particularly abundant and species-rich in the polar regions, where genetic studies have identified several cases of unrecognized diversity [3, 4].

Although pycnogonids are widespread in all oceans and have been known to science for over 250 years, the feeding habits of most taxa remain poorly studied and a detailed review on the feeding ecology of pycnogonids has, to our knowledge, never been published. Observations on this topic are generally scattered throughout the literature, and especially publications written in languages other than English are often difficult to find. General textbooks usually only state that pycnogonids feed mostly on sessile prey, such as coelenterates, sponges and bryozoans (e.g., [5]).

In the present paper, we review all available observations published in the last two centuries including both detailed studies and preliminary notes, thus providing a state of the art summary of known food preferences for this bizarre and highly understudied group of exclusively marine arthropods. Additionally, we discuss morphological correlates of different feeding preferences and the occurrence of generalism vs. specialization in various pycnogonid taxa.

## Morphological features for food uptake

**Fig. 1:**
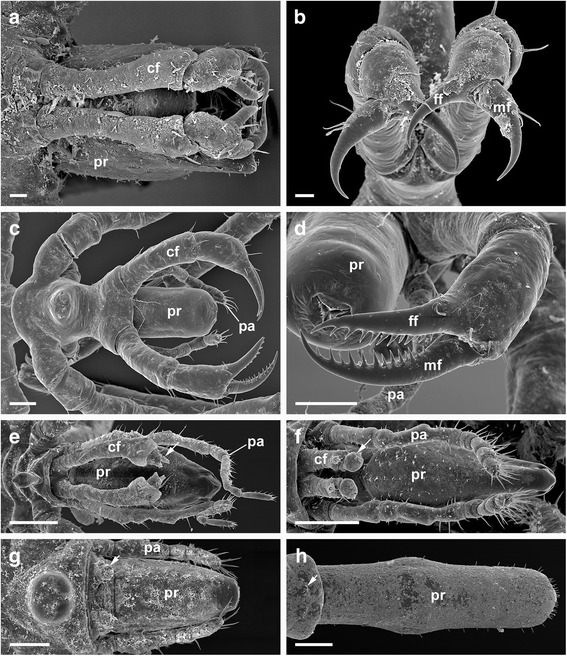
Chelifores and palps of different pycnogonid families showing different morphologies. Originals, except B after [115]. **a** *Anoplodactylus angulatus*, with dorsally positioned chelifores, palps absent. Bar 20 μm. **b** *Anoplodactylus petiolatus*, detail of chelifore with unarmed fixed and movable finger. Bar 20 μm. **c** *Nymphon gracile*, with laterally positioned chelifores and dorsally positioned palps. Bar 100 μm. **d** *Nymphon gracile*, detail of chelifore with toothed fixed and movable finger. Bar 100 μm. **e** *Ammothella appendiculata*, with reduced chela. Fixed and movable finger still present (arrow). Palps long, extending beyond proboscis. Bar 200 μm. **f** *Achelia echinata*, with reduced chela. Fixed and movable finger fused to small bud (arrow). Palps with approx. Same length of proboscis. Bar 200 μm. **g** *Tanystylum conirostre*, chelifore reduced to small bud with seta (arrowhead). Palps shorter than proboscis. Bar 100 μm. **h** *Endeis spinosa*, chelifore reduced protuberance with seta (arrowhead). Palps absent. Bar 200 μm. cf., chelifore; ff, fixed finger; mf, movable finger; pa, palpus; pr, proboscis

**Fig. 2:**
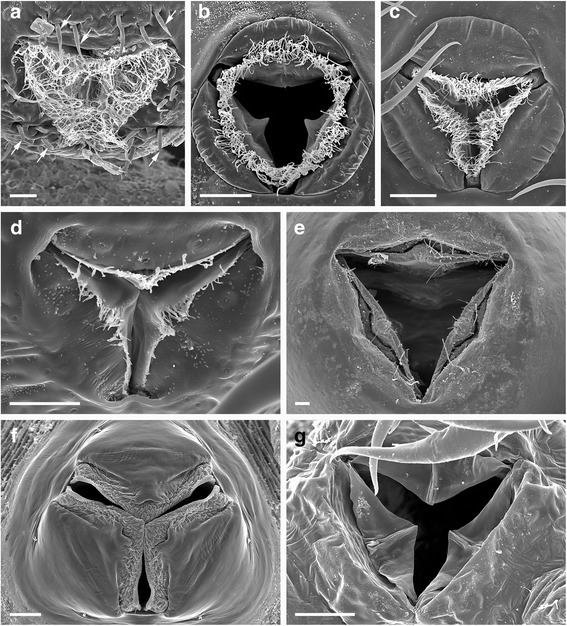
Mouth openings of different pycnogonid families showing different morphologies. Dorsal is up. Originals, except A, B, C, F, G after [119]. Bars 20 μm. **a** *Endeis spinosa,* mouth surrounded by setae (arrows) and lips fringed with many microtrichia. **b** *Callipallene tiberi,* mouth closed, lips fringed with microtrichia. **c** *Callipallene phantoma*, mouth open, lips fringed with microtrichia. **d** *Nymphon gracile*, lips fringed with few microtrichia. **e** *Pycnogonum littorale*, lips occasionally fringed with microtrichia. **f** *Ammothella appendiculata*, mouth without seta or microtrichia, but fringed with papillae. **g** *Anoplodactylus angulatus*, mouth equipped with three valves

A pycnogonid that features all appendages used for feeding (*Nymphon gracile*) is pictured in Fig. 1c. As the main organ for food uptake, pycnogonids have a unique triradially symmetric proboscis with a terminal mouth surrounded by three movable lips and gland openings probably secreting saliva [6]. The proboscis musculature allows suction and pumping of food, mostly in liquid form. Moreover, the proximal part of the proboscis contains the pharyngeal filter, also termed “oyster basket” or “Reusenapparat” (in old literature in German, e.g. [7]), which is composed of densely packed bristles that are used to filter out or grind ingested solid particles. Recently Wagner et al. [8] have compared pharynx inner surfaces of various pycnogonids using scanning electron microscopy and showed taxon-specific features of the filter bristles and other pharynx armatures, e.g. denticle arrays. However, as differences in feeding ecology between pycnogonid taxa are so far poorly known, no definite conclusions on correlation with feeding modes could be made. The morphology of the mouth opening also differs, as the lips are often fringed with microtrichia of various numbers and lengths (Fig. 2). In some cases, these are reduced or lost, and the lips are either fringed with papillae (some ammotheids) or not armed at all, as in *Anoplodactylus*. Pycnogonid taxa also differ in whether the mouth is surrounded by setae, as in *Endeis* (Fig. 2a), or not, as e.g. in *Ammothella* (Fig. 2f). In *Endeis*, which lacks palps, the setae have a tactile function [1]. This indicates that different pycnogonid taxa have different “toolboxes” for handling food, though in a superficial inspection the general morphology of their feeding apparatus looks quite uniform. Ammotheids and ascorhynchids, most of which lack functional chelifores and feed on hydroids, often have a more mobile proboscis than nymphonids and other taxa with chelifores [1]. However, this does not apply to taxa without chelifores that are parasitic on much larger animals (Pycnogonidae) or detritivorous (*Endeis*).

Other organs important for feeding in pycnogonids are the chelifores and palps, which are homologous to the arachnid chelicerae and pedipalps, respectively [9]. The chelifores consist of a scape and a chela with a movable and an immovable finger and are used for cutting off and macerating pieces of the prey organism and leading them to the proboscis (Fig. 1). The chelifores can be placed dorsally (e.g. in the Phoxichilidiidae, Fig. 1a,b) or laterally (e.g. in *Nymphon*, Fig. 1c,d) of the proboscis. According to Wyer & King [10], only species with laterally positioned chelifores use them to macerate prey, as they are more mobile than dorsally placed ones. For this purpose, when the chelifores are laterally positioned, they often have serrated chelae (Fig. 1d). In the adults of some taxa, the chelifores are highly reduced (many Ammotheidae, Fig. 1e-g) or lost (Austrodecidae, Colossendeidae, Rhynchothoracidae, Pycnogonidae, Endeidae, Fig. 1h). The palps are, besides their tactile function, also used to hold the prey items or guide the proboscis. Palps differ between taxa in the degree of robustness and supination as well as in their length relative to the proboscis and the number and proportion of articles (Fig. 1e-g). In some taxa they are reduced or lost (Pycnogonidae, Callipallenidae, Pallenopsidae, Phoxichilidiidae, Endeidae, Fig. 1a,h). The walking legs, of which there are four (rarely five or six) pairs, can also be used to hold prey, and the morphology of their distal parts also differs between taxa. The prey is held between the claw and the propodus, which often has spines on its ventral surface. In some pycnogonids, such as *Nymphon brevirostre* and members of the Phoxichilidiidae, the tarsus is extremely short and the propodus is curved, apparently as an adaptation for climbing among hydroids, on which they feed [11].

The digestive system of pycnogonids was described by Fahrenbach & Arango [6]. It is divided into a foregut within the proboscis, where food processing and filtering take place as described above, a midgut where the food is digested and absorbed, and a hindgut covered by cuticle in the reduced abdomen. The midgut is remarkable in that it has diverticula extending into the walking legs and chelifores, which in most, but not all species reach almost to the tips of these appendages. The mechanism of digestion was described by Richards & Fry [12]. Intracellular digestion occurs exclusively by pinocytosis, i.e. only liquid material is taken up.

## What do sea spiders eat?

**Fig. 3:**
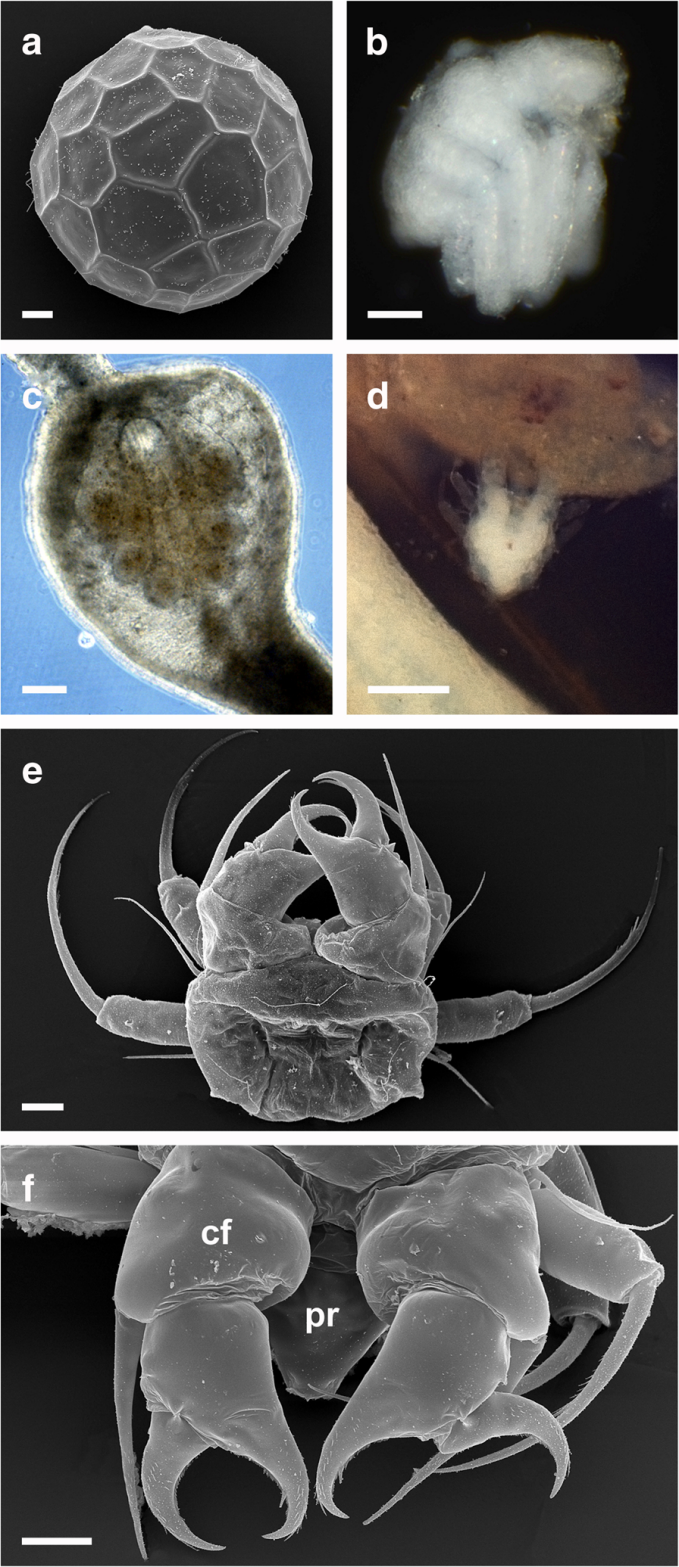
Feeding and morphological features of protonymphon larva and subadults. Originals, except C after [42]. **a** *Callipallene spectrum*, SEM micrograph of Egg. Bar 20 μm. **b** *Callipallene producta*, newly hatched postlarva. Bar 40 μm. **c** *Anoplodactylus petiolatus*, larva in a gallzooid of *Hydractinia echinata*. Bar 50 μm. **d** *Achelia spec*., protonymphon detached on host organism. Bar 100 μm. **e** *Achelia echinata*, SEM micrograph of protonymphon, dorsal view. Bar 20 μm. **f** *Achelia echinata*, SEM micrograph of chelifore and proboscis. Bar 20 μm. cf., chelifore; pr, proboscis

Pycnogonids are usually described as predatory or parasitic. The difference between these terms is that, while predators kill their prey and often consume all or most of the organism, parasites usually do not directly kill their host [13]. Under this definition, most pycnogonids can be described as parasitic. Parasitism in pycnogonids was reviewed by Staples [14], who also treated feeding on hydroids and other colonial organisms as parasitism, not as predation. While infestations occasionally lead to the death of the host (e.g. [15]), this also occurs in other parasite-host relationships. However, there are some cases of predation by pycnogonids, in which entire animals (e.g. annelids; [16, 17]) were consumed. In almost all cases, parasitism by adult pycnogonids can be categorized as ectoparasitism, although some instances of endoparasitism in the pallial cavity of molluscs and in actinians are known. Other pycnogonids can be described as herbivorous [10] or detritivorous (e.g. [17]). Pycnogonid larvae are either obligate parasites or lecithotrophic and can be either ecto- or endoparasitic (see overview in [18]). Chelifores, palps and ovigera are already present in the earliest larval stages and are used for attachment to the host (Fig. 3d-f).

## Feeding specializations

In the following section, published records of feeding by pycnogonids on different types of prey are summarized (see also Table 1) and possible specializations of various taxa are discussed.

**Table 1:** Summary of known food sources for pycnogonid family-level taxa

	Algae	Sponges	Hydroids	Actinians	Corals	Medusae	Bryozoans	Mollusks	Annelids	Crustaceans	Echinoderms	Detritus
Austrodecidae			+				+					
Colossendeidae		?	+	+	+	+		+	+			+
Rhynchothoracidae			+				+					
Pycnogonidae				+		+	+				+	+
Ascorhynchidae		+			?			+				
Nymphonidae	?		+	+	?		+	+	+	+		+
Callipallenidae			+				+		+			
Pallenopsidae			?		+	+						+
Phoxichilidiidae		+	+			+	+	+	+	+	+	
Endeidae			+	+	+							+
Ammotheidae	+	+	+	+	+	+	+	+	+	+	+	+
Incertae sedis				+					+			

A plus sign indicates a definitive feeding association, a question mark indicates an association not confirmed by direct observations of feeding or gut content

### Algae

Zenker [19] reported about finding tissue of probably brown algal origin in the proboscis of *Nymphon gracile*. Wyer & King [10] mentioned *Ammothella longipes* feeding on the red alga *Mastocarpus stellatus*. In the case of *A. longipes* on brown algae (*Halopteris*), Soler-Membrives et al. [17] found this species not actually consuming the algae, but the detritus accumulated on them. Bamber & Davis [20] showed that *Achelia echinata* feeds on the green alga *Ulva* and the red alga *Griffithsia* by labelling the algae radioactively. *Ulva* seems to be preferred. From the paucity of observations, we conclude that algae or detritus from algal structures seem to be a food source of minor importance, although they are consumed by several phylogenetically distantly related sea spiders. It is possible that algal tissue is sometimes ingested when pycnogonids are feeding on organisms living on the algae, or as part of the gut content of their prey.

### Sponges

Marcus [21] observed a specimen of *Ascorhynchus corderoi* feeding on an unidentified sponge. Dayton et al. [22] recorded *Ammothea striata* feeding on a sponge, which also was not identified. *Colossendeis* was observed carrying a piece of possible sponge underneath its body [23]. Cuartas & Excoffon [24] reported that *Tanystylum orbiculare* and *Anoplodactylus petiolatus* fed on the demosponge *Hymeniacidon perlevis* when their preferred hydroid prey was not available. In conclusion, sponges appear to be uncommon as a pycnogonid food source, although they are often mentioned as such in more general reviews. However, it should be noted that pycnogonid feeding on sponges is understudied, as most of the studies investigating food preference in pycnogonids did not include sponges as a possible prey item (e.g. [25]). The results of the only study known to us that does include them [26] were inconclusive as to whether the pycnogonids actually fed on the sponges.

### Hydroids

Associations of pycnogonid larvae with their (mostly hydroid) hosts have been summarized by King [1] and Staples & Watson [27]. The larvae of some phoxichilidiids and ammotheids are endoparasites forming galls in the gastral cavity of hydroid polyps. Hodge [28] first observed this for *Phoxichilidium femoratum* on *Coryne eximia* and Semper [29] documented the development of the same species in more detail on *Hydractinia echinata*. Dogiel [30] also found a similar mode of development in *Endeis spinosa*, whose larva develops attached to the hydranth of *Obelia* sp. Since then, such a relationship has also been found in many other species (see overview in [31]). In most Ammotheidae and Pycnogonidae as well as in *Nymphon gracile* [32], the larvae are ectoparasites of hydroids, although in the Pycnogonidae the adults feed mostly on actinians [30, 33]. Russel & Hedgpeth [34] reported on the presence of larvae of two ammotheid species on the hydroid *Orthopyxis everta*, the ectoparasitic *Ammothea hilgendorfi* and the endoparasitic, gall-forming *Tanystylum duospinum*. Adults of both species are also found on the hydroid. Often the larvae appear to be host-specific and development can differ even between closely related species, e. g. *Anoplodactylus pygmaeus* larvae form galls in the gastral cavity of *Obelia* polyps, while those of the closely related *A. petiolatus* live attached to the manubrium of medusae from the same genus [35].

Feeding of adult pycnogonids on hydroids also has often been documented. Cole [36] observed adults of *Anoplodactylus lentus* feeding on *Eudendrium ramosum*. The hydranths were cut off with the chelifores and placed in front of the mouth. Loman [37] reported the same for *Phoxichilidium femoratum* feeding on *Tubularia*, with gonophores being preferred as food over other parts of the hydroid. According to Loman [38], *Nymphon brevirostre* feeds on the same species. Prell [39] reported that several *Nymphon* species from the North Sea feed almost exclusively on thecate hydroids (*Lafoea* in the wild, *Campanularia* in an aquarium setting). The hydrothecae are led to the mouth without breaking them off using the chelifores. Athecate hydroids are consumed only in case of extreme starvation. Agreeing with this, according to Schlottke [40], *N. brevirostre* prefers the thecate *Obelia geniculata* to the athecate *Coryne pusilla*. He also observed *Anoplodactylus pygmaeus* and *Phoxichilidium femoratum* feeding on various hydroid species. Wyer & King [10] observed several species of North Atlantic pycnogonids (*Nymphon gracile*, *Phoxichilidium femoratum*, *Anoplodactylus petiolatus* and *Achelia echinata*) feeding on *Dynamena pumila*, while *Nymphon brevirostre* fed on various hydroids epizoic on the bryozoan *Flustra foliacea*. They noted that in *N. gracile* the (laterally positioned) chelae were used to macerate the prey whereas this is not the case in the phoxichilidiids, where they are dorsally positioned and only used for grasping. *A. echinata*, which has reduced chelifores, grasps hydroid tentacles and pulls them off with the proboscis lips. Lotz [16] found that *Achelia echinata*, *Nymphon brevirostre* and *Callipallene brevirostris* do not accept non-hydroid food, and starve if no hydroids are present. However, *Anoplodactylus petiolatus*, which normally also feeds on hydroids, does accept other food. Stock [25] showed *Nymphon gracile*, *N. brevirostre* and *Endeis spinosa* are chemically attracted to various hydroid species. While *N. brevirostre* and *E. spinosa* prefer *Laomedea*, *N. gracile* prefers *Dynamena*. Staples & Watson [27] documented multiple cases of pycnogonid-hydroid association in Australia and New Zealand. Particularly notable is the association of *Austrodecus frigorifugum* with *Dictyocladium monilifer*. The pycnogonid, which lacks chelifores, inserts its very narrow proboscis, guided by its palps, into the hydrothecae and gonothecae of the hydroid. In contrast, the related Antarctic species *A. glaciale* feeds mostly on bryozoans [26]. According to Staples & Watson [27], the pointed proboscis of *Achelia transfugoides* is adapted for feeding on the hydrothecae of *Stereotheca elongata* and *Sertularia marginata*. They also report that *Parapallene australiensis* occurs in such great numbers on *Halopteris glutinosa* that they infer an obligatory association, and the same appears to be the case for *Tanystylum* sp. and *Pennaria wilsoni*. According to Varoli [41], both *Anoplodactylus stictus* and *Tanystylum isabellae* accept *Sertularia* as food, but not *Dynamena*. Both hydroids belong to the family Sertulariidae. Heß & Melzer [42] reported on the feeding of *Anoplodactylus petiolatus* on *Hydractinia echinata*. The pycnogonid feeds mostly at night and avoids touching the hydroid polyps, feeding mostly on the tips of spines. However, even pycnogonids that are almost completely engorged by the polyps are able to pull themselves out using their legs.

**Fig. 4:**
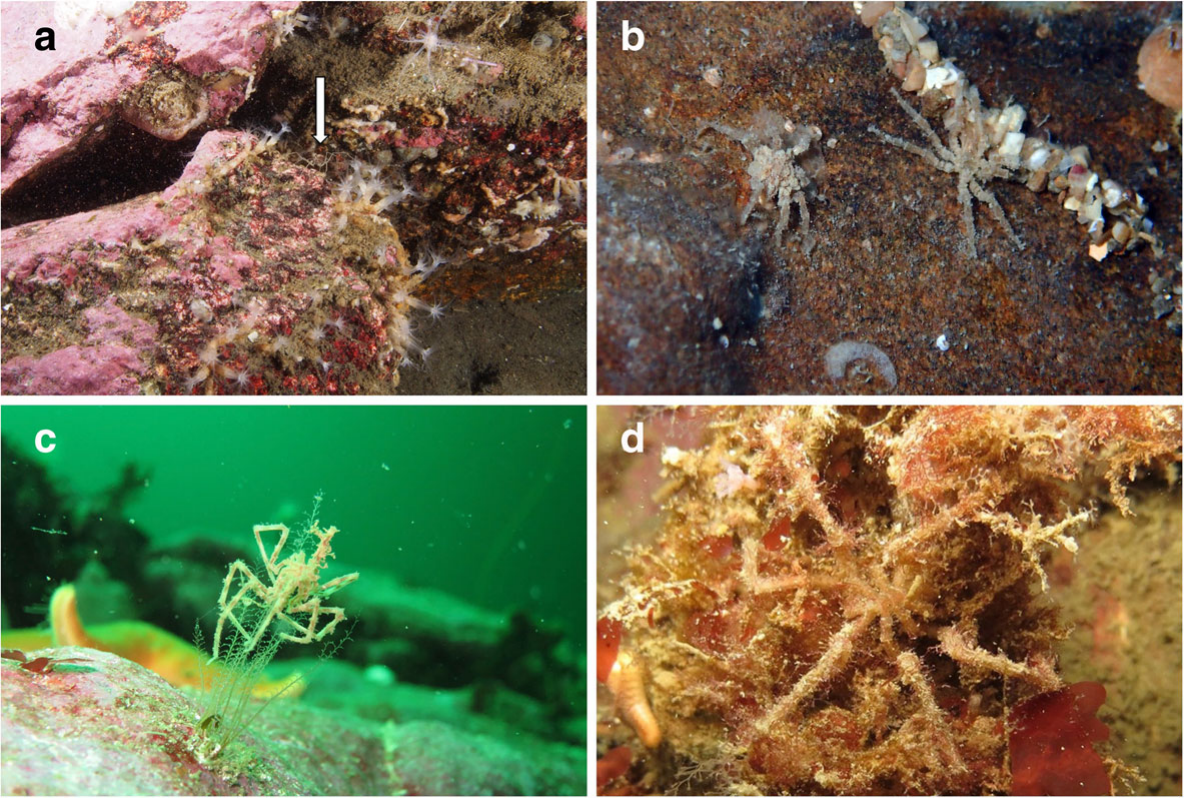
Pycnogonids in their natural environment, near possible food sources. **a** *Callipallene margarita* and its surroundings mainly built-up by red algae, *Clavularia* octocorals, and organic debris; Southern Chilean fjords, photo: Kaitlin McConnell. Pycnogonid indicated by arrow. **b** Female (right) and male (left) *Achelia langi* under a stone in wave dominated upper infralitoral near a *Polycirrus* polychaete; note male carrying fertilized eggs; Northern Adriatic, photo: Roland Melzer. **c** and **d** *Pallenopsis yepayekae*; C on a plumulariid hydrozoan. The pycnogonid may be feeding on the polyps, but this cannot be certainly determined. Southern Chilean fjords, photo: Roland Meyer. D On red algae, well camouflaged by a “roof-garden”. Southern Chilean fjords, photo: Roland Melzer

As pycnogonids are particularly common in the Southern Ocean, many observations of their feeding on hydroids are also recorded from there. Hodgson [43] mentioned that the Antarctic pycnogonid *Decolopoda* was observed holding *Tubularia* hydranths in its chelae (note that *Decolopoda* and *Dodecolopoda* are unusual among the Colossendeidae by their presence of chelifores). According to Dayton et al. [22], *Colossendeis robusta* and *C. megalonyx* were also seen feeding exclusively on hydroids, mostly on a small unidentified species growing on sponges. An unidentified species of *Colossendeis* was also photographed feeding on a solitary hydroid in the North Central Pacific [44]. Fry [26] found that, when provided with a diverse selection of food items, *Rhynchothorax australis* preferred hydroids, especially *Eudendrium tottoni*. The preference of *R. australis* for *E. tottoni* was explained by the fact that this was the only athecate among the tested hydroid species, and that its hydranths are therefore less protected. However, this explanation seems to be contradicted by the observation that *Nymphon* follows the opposite pattern [39]. *Austrodecus glaciale* also fed on hydroids, although its preferred food was bryozoans. Stout & Shabica [45] also recorded several other Antarctic species (*Austrodecus* sp., *Pentanymphon antarcticum*, *Nymphon* sp., *Achelia* sp.) associated with or feeding on hydroids. Richards [46] reported that *Nymphon australe* was found with hydroid colonies grasped in its chelifores. *Pallenopsis yepayekae* was photographed on a plumulariid hydrozoan (this paper, Fig. 4c), but it cannot be determined whether feeding actually took place. In conclusion, hydroids seem to be a food item of major importance for most pycnogonid groups. We found more records of pycnogonids feeding on hydroids than on any other type of prey. It is possible that, in some cases, pycnogonids attack hydroids to feed on their gut content, as has been observed for sea anemones (see below). This behaviour would be a type of kleptoparasitism, or if the hydroid is also consumed, kleptopredation, as has been observed in nudibranchs [47].

### Actinians

Pycnogonids in the family Pycnogonidae appear to be specialist feeders on actinians. The wide proboscis and the ability to open the mouth widely can be interpreted as specializations for ingesting large amounts of soft-bodied animal tissue. Although associations between Pycnogonidae and anemones had been observed earlier, the feeding mechanism of *Pycnogonum* was first documented by Prell [39] for *P. litorale* on *Metridium* and *Urticina crassicornis*. According to him, the animal feeds mostly on the pedal disk of the actinians, using its first pair of legs to span the skin before inserting its proboscis (Pycnogonidae lack chelifores and palps). The same was observed by Wyer & King [10] for *P. litorale* feeding on various actinian species. Arndt [48] reported an individual of the same species with its proboscis bored into a tentacle of *Edwardsiella loveni*. Wilhelm et al. [33] documented that, after the transition from larval to juvenile stage, *P. litorale* immediately shifts from its original hydroid host to the actinian *Metridium senile*. Bamber [49] showed that *P. litorale* had a preference for some anemones (*Calliactis* and *Adamsia*) over others (*Actinia* and *Tealia*). In the case of *Adamsia,* the entire anemone was consumed. These observations are difficult to explain as the preferred anemones are symbionts of hermit crabs and therefore normally inaccessible to the pycnogonids. Other species of *Pycnogonum*, such as *P. stearnsi* [26] and *P. benokianum* [50] have also been documented as actinian predators.

Other pycnogonids have also been documented feeding on actinians. Stock [25] showed that *Nymphon brevirostre*, and possibly *Endeis spinosa*, can discern the presence of actinians in seawater by chemical cues and are attracted to them, although they are not the preferred food. *Artemidactis victrix* is the preferred food of *Ammothea striata* according to Stock [22]. Wyer & King [10] reported *Nymphon gracile* feeding on *Actinia equina*. In most cases, the feeding mechanism was similar to that of *Pycnogonum*, but occasionally tentacles or other pieces of the actinian were removed with the chelifores. Richards [46] observed *Ammothea carolinensis* feeding exclusively on anemones. *Nymphon orcadense*, *N. hirtipes* and *Decolopoda australis* were also observed feeding on actinians in an aquarium setting. *A. carolinensis* inserted its proboscis into the mouth opening of the anemone, leading to the suggestion that it feeds only on the gut contents (kleptoparasitism). *D. australis* was observed carrying the anemone around in its proboscis after separating it from the rock. This behavior is also visible in a photograph by Wu [51] showing an Antarctic pycnogonid identifiable as belonging to the *Colossendeis megalonyx* complex. Braby et al. [52] observed *Colossendeis minuta* and *C. colossea* feeding on the anemones *Anthosactis pearseae* and *Liponema brevicorne*. While the smaller *A. pearseae* was always consumed in its entirety after separating it from the rock, in *L. brevicollis* sometimes autotomized tentacles were consumed. *Colossendeis* sp. was also observed feeding on actinostolid anemones in the Southern Ocean [53]. Mercier et al. [54] also observed *N. hirtipes* feeding on the actinian *Stephanauge nexilis* in the wild. Mercier & Hamel [15] reported on the small pycnogonid *Pigrogromitus timsanus* parasitizing the actinian *Bartholomea annulata*, leading to the host’s death. The pycnogonids were found more frequently on the column than on the tentacles, which would enable them to feed on the gonads. This agrees with other observations (e.g. [37]) that pycnogonids preferentially feed on the gonadal tissues of coelenterates. Endoparasitism of actinians (*Entacmaea quadricolor*) by juvenile pycnogonids (*Ammothella biunguiculata*) has also been documented [55]. Therefore, actinians are an important food source mostly for members of the Pycnogonidae, as well as some pycnogonids belonging to other taxa.

### Other cnidarians

Pycnogonids have also been documented to feed on medusae of various taxa. Prell [39] mentioned *Pycnogonum litorale* feeding on the stauromedusa *Lucernaria*. *Phoxichilidium femoratum* also fed on *Lucernaria*, cutting off branched tentacles with the chelifores. A similar technique is used by other species, although younger larvae appear to use their chelifores only for clinging to the host [10]. Uchida & Hanaoka [56] reported ammotheids feeding on the stalked medusa *Manania distincta*. An unidentified species of *Colossendeis* was photographed feeding on a coronate medusa in the North Atlantic [44]. *Colossendeis* was also observed feeding on medusae entrapped by sea anemones (Moran, pers. comm. cited by [44]). Lebour [57] found larvae of *Anoplodactylus petiolatus* on five different species of medusa, most frequently on *Obelia* sp.. Wyer & King [10] reported larvae of the same species from the medusa *Clytia hemispherica*. Okuda [58] recorded larvae of *Achelia alaskensis* developing on the hydromedusa *Polyorchis karafutoensis*. Mauchline [59] found unidentified juvenile pycnogonids attached to the medusa *Periphylla periphylla*, and Child & Harbison [60] recorded both adults and juveniles of *Bathypallenopsis scoparia* from the same species. Examination of the gut contents suggested that the adult had eaten the tentacles, but the juveniles fed on the gonads or the contents of the gastrovascular sinus. Similarly, Pagès et al. [61] reported *B. tritonis* attached to *Pandea rubra*. *Bathypallenopsis calcanea* was found on the medusa *Aeginura grimaldii*, but no evidence of feeding by the pycnogonid was observed [62]. Other species of pycnogonids found in bathypelagic samples (*Bathypallenopsis* spp. and *Colossendeis gardineri*) are probably also associates of medusae or other pelagic organisms [63]. Unlike some other animals associated with medusae, e.g. some copepods [64], the morphology of these pycnogonids does not appear to be greatly modified.

There have been several reports of pycnogonids associated with corals, e.g. *Boehmia chelata* and alcyonarians [65]. Stephensen [66] noted that *Nymphon hirtipes* is only found where the soft coral *Eunephthya* occurs, while *Boreonymphon robustum* is probably associated with *Umbellula encrinus*. He noted that the peculiar shape of the *Boreonymphon* chelae may be adapted to grasping *Umbellula* tentacles, and specimens carrying juveniles were often found in places with smaller coral species. The ammotheid *Tanystylum grossifemorum* has been recorded from several octocoral species [67]. Child [68] found several species associated with the scleractinian coral *Oculina varicosa*. In none of these cases, pycnogonids were directly observed feeding on the corals. However, corals are known to be hosts of pycnogonid larvae. Moseley [69] found cysts containing unidentified pycnogonid larvae in the gastric cavity of gastrozooids of the hydrocoral *Pliobothrus symmetricus*. Stock [70] described galls containing larvae probably belonging to *Ascorhynchus* in the soft coral *Chrysogorgia papillosa*. Feeding of adult pycnogonids on corals was to our knowledge first reported by Slattery & McClintock [71], who found *Colossendeis megalonyx* to feed on the soft corals *Alcyonium antarcticum* and *Clavularia frankliniana*. *Colossendeis robusta* was also found feeding on the latter species, while *Ammothea* sp. fed on *Gersemia antarctica*. Arango [72] recorded *Endeis mollis* feeding on the hydrozoan coral *Millepora exaesa* and the zoanthid *Palythoa caesia* and *E. biseriata* feeding on the zoanthid *Protopalythoa* sp. A pycnogonid probably identifiable as *Bathypallenopsis mollissima* has been observed feeding on an unidentified bamboo whip coral (Isididae) according to Watling et al. [73]. Feeding of adult pycnogonids on corals, therefore, appears to be little documented, although it may be especially common in deep-sea forms.

### Bryozoans

Predation of pycnogonids on bryozoans has been reviewed by Ryland [74] and Key et al. [75]. Prell [39] mentioned, without further details, *Phoxichilidium femoratum* feeding on the bryozoan *Crisia*. Fry [26] found that both *Austrodecus glaciale* and *Rhynchothorax australis* fed on all five bryozoan species that were presented to them, but they were not among the preferred foods of *Rhynchothorax*, while *Austrodecus* showed a strong preference for the bryozoan *Cellarinella roydsi*. He pointed out that the extremely thin distal proboscis of austrodecids appears to be an adaptation for feeding on bryozoan zooids through the frontal wall pores. *Cellarinella roydsi* is the only one of the tested bryozoan species that has numerous frontal pores. However, according to Ryland [74], it is also possible that the pycnogonid feeds through the peristome, as the species does not have an operculum. Most of the pores also do not penetrate the entire frontal wall [76]. The spiny palps of *Austrodecus* are probably used to guide and strengthen the proboscis [77]. Wyer & King [10, 78] recorded *Achelia echinata* feeding on *Flustra foliacea*, inserting the proboscis through the operculum. However, *Ammothella longipes* would not feed on the bryozoans even when the zooids were extended, instead preferring the red algae growing on the bryozoan colony. *Pycnogonum litorale* was observed feeding on the rotting edge of a colony of the same species. *Nymphon gracile* was observed feeding on *Amathia imbricata*, using the same method as on hydroids. Varoli [41] reported that both *Anoplodactylus stictus* and *Tanystylum isabellae* would feed on *Amathia distans*. Sherwood et al. [79] showed that *Stylopallene longicauda* sequesters amathamine alkaloids from *Amathia wilsoni*, therefore demonstrating that this bryozoan is a food source of the pycnogonid. The alkaloids are probably used as a chemical defense. According to Staples [80], the digitiform chelae of *Pseudopallene watsonae* larvae are probably used to manipulate the manubrium of bryozoan zooids before inserting the proboscis. In the adult, however, the chelae are robust as in other species of *Pseudopallene* and appear more suited to crushing bryozoan zooids. It, therefore, appears that bryozoans are an important food source for many different pycnogonid taxa, and bryozoan feeders often show clear specializations such as an extraordinarily thin proboscis or chelifores suitable for crushing.

### Mollusks

Parasitism of pycnogonids on mollusks was reviewed by [81]. Merton [82] recorded a nymphonid, which he named *Nymphon parasiticum*, parasitic on the nudibranch *Tethys fimbria*. However, no fully grown specimen was found, and the species was to our knowledge never recorded again. Similarly, Ohshima [83] recorded a juvenile ammotheid parasitic on the nudibranch *Armina variolosa*. Stock [68] recorded a juvenile of an unidentified species of *Ascorhynchus* parasitic on the gills of the nudibranch *Aplysia dactylomela*. Edmunds [84] found unidentified pycnogonids feeding on the nudibranchs *Cuthona perca* and *Spurilla neapolitana*. In one case the proboscis was inserted into the liver duct. Piel [85] reported *Anoplodactylus californicus* preying on the nudibranch *Dondice occidentalis*, grabbing cerata with the chelicerae, causing ceratal autotomy and consuming them. Rogers et al. [86] observed that *Anoplodactylus evansi* consumed 13 different species of opisthobranchs in an aquarium setting. The species would consume almost no other prey that was offered. Whole animals were consumed after immobilizing them with the claws of the front legs. Arango & Brodie [87] recorded *A. longiceps* preying on the nudibranch *Okenia* sp., and Mercier et al. [51] reported about a specimen of *Nymphon hirtipes* feeding on a nudibranch (*Tritonia* sp.), which was shredded and ingested completely.

Pycnogonids have also been recorded feeding on shelled gastropods. Shabica [88] mentioned *Colossendeis megalonyx*, *C. robusta* and *Pentanymphon* sp. as predators of the Antarctic limpet *Nacella concinna*, and Bain [89] observed *Anoplodactylus californicus* feeding on the prosobranch snail *Pleurobranchus digueti*. The species *Ascorhynchus endoparasiticus* is parasitic in the pallial cavity of the opisthobranch *Scaphander punctostriatus* [90].

Bivalves are also known to be a food source for pycnogonids. The ascorhynchid *Nymphonella tapetis* is an economically important parasite of various bivalve species in the Northwest Pacific [91]. Only juveniles are parasitic. Curiously, in other *Nymphonella* species, which may be synonymous with *N. tapetis*, endoparasitism has never been recorded [92]. *Nymphonella* is phylogenetically nested within *Ascorhynchus*, which includes other mollusk-feeding species [92]. Arnaud & Bamber [2] reported the presence of juveniles of two different unidentified *Ascorhynchus* species as endoparasites in *Tellina perna*. Benson & Chivers [93] recorded an infestation of the mussel *Mytilus californianus* by the normally free-living species *Achelia chelata*. Tharme et al. [94] reported an unidentified pycnogonid, represented by larvae as well as adults, living parasitically on the bivalve *Donax serra*. Lotz [16] mentioned that *Anoplodactylus petiolatus* would consume *Mytilus* tissue when the preferred food was not available. The same was observed by Bain [89] for *A. californicus* and by Varoli [41] for *Tanystylum isabellae*. While mollusks can be consumed by a variety of pycnogonid taxa, only a few species, mostly ascorhynchids, are specialized molluscan parasites.

### Annelids

While there are several records of pycnogonids on tubicolous polychaetes (e.g. [45]), it was not clarified whether they feed on the polychaetes themselves or on their epibionts. However, Wyer & King [10] recorded *Nymphon gracile* feeding on an unidentified sedentary polychaete. Richards [46] recorded that an unknown sedentary polychaete living on red seaweed seemed to be the preferred food of the Southern Ocean species *Nymphon orcadense*, and was also accepted by starved specimens of *N. australe*. *Nymphon molleri* was observed feeding on the spionid polychaete *Polydorella stolonifera*, *Anoplodactylus evansi* on an unidentified small polychaete and *Ammothea australiensis* on the tubicolous polychaete *Galeolaria caespitosa* [95]. The latter species prevented the polychaete from retracting by placing its palps behind the branchial crown and operculum. Shabica [96] recorded *Colossendeis megalonyx* feeding on tubicolous polychaetes in a tank setting. *Achelia simplissima* feeds on the spirorbid *Spirorbis bifurcatus* [97]. Salazar-Vallejo & Stock [98] recorded the larvae and juveniles of a pycnogonid tentatively identified as *Ammothella spinifera* developing on *Sabella melanostigma*. The abdominal segments of the host, which contain the reproductive tissue, were preferred to the thoracic ones.

Pycnogonids have also repeatedly been reported to feed on errant annelids. Hilton [99] recorded a callipallenid identified only as “*Pallene*” “devouring a soft annelid worm”. Similarly, Lotz [16] recorded *Anoplodactylus petiolatus* eating errant polychaetes in an aquarium setting, fully ingesting them. Rogers et al. [86] also found *A. evansi* eating an unidentified errant polychaete. Stock [100] recorded a juvenile, tentatively referred to *Hannonia* (a genus of uncertain placement) as parasitic on the polychaete *Cirriformia capensis*. *Ammothella longipes* was recorded feeding on nereid polychaetes [17, 101]. The species appears to be carnivorous during spring and summer and detritivorous in the winter based on fatty acid analyses [102]. It appears that annelids are a food source of medium importance used by many different species but there are few annelid specialists.

### Crustaceans

Richards [46] mentioned that *Nymphon orcadense*, in the absence of its preferred polychaete food, would consume dead amphipods. Lotz [16] reported that, in the absence of its favored food source (hydrozoans), *Anoplodactylus petiolatus* would catch and eat copepods of the species *Tisbe furcata*. When a copepod touches the pycnogonid’s body, it is caught with the claw of a walking leg. It is then placed in front of the proboscis opening first using the claws of both legs of a pair and then using the chelifores, before being sucked out. Bain [89] reported *Anoplodactylus californicus* feeding on brine shrimp (Anostraca), which were caught directly from the water column with the chelifores. Varoli [41] reported that dead specimens of the amphipods *Apohyale media* and *Caprella danilevskii* and the anostracan *Artemia salina* were accepted by *Anoplodactylus stictus* and *Tanystylum isabellae*, but living ones were not. Soler-Membrives et al. [17] recorded *Ammothella longipes* holding caprellid amphipods, but it was not observed whether they were actually feeding on them. Thus, crustaceans seem to be a food source only in few cases, and probably mainly dead amphipods or copepods are important in that respect.

### Echinoderms

Stock [102] described the species *Pycnosomia asterophila*, which was found only on the oral surface of the asteroid *Calliaster corynetes*. Nakamura & Fujita [103] found juveniles and adults of *Ammothea hilgendorfi* on *Coscinasterias acutispina*, mostly on the aboral and lateral surfaces.

Sloan [104] recorded the species *Anoplodactylus ophiurophilus*, which is exclusively found attached to the oral side of ophiuroids of the genus *Ophiocoma*. The species *O. doederleini* appears to be preferred. The pycnogonid evidently feeds on the oral mucus which the ophiuroids produce to entrap particles.

Losina-Losinsky [105] found specimens of *Pycnosomia strongylocentroti* attached to the spines and pedicellariae of an echinoid (*Strongylocentrotus*) with their legs. He noted that the propodus of this species appears specialized for such an attachment.

Prell [39] reported one case where *Pycnogonum litorale*, which is normally specialized on actinians, fed on the holothurian *Cucumaria frondosa*. Ohshima [106] reported juveniles of *Ammothella biungiuculata* and *Ammothea hilgendorfi* associated with the holothurians *Apostichopus japonicus* and *Holothuria lubrica*, respectively, although actual feeding was not observed. Echinoderms, therefore, seem to be a food source of minor importance, which is used mostly by a few specialized phoxichilidiid species.

### Sediment and detritus as a food source

Pycnogonids have also been observed as sediment feeders. Stout & Shabica [45] recorded the Antarctic species *Decolopoda australis* and *Pallenopsis* cf. *patagonica* “feeding in the soft sediments”. Similarly, photographs of Antarctic *Colossendeis* specimens with their proboscis inserted into sediment led Hedgpeth [107] to conclude that these animals feed on the meiofauna living in the uppermost sediment layers. While this seems likely in this case, pycnogonids were also observed to feed on organic detritus. Wyer & King [10] observed starved specimens of *Nymphon gracile* feeding on the detritus that had accumulated on their bodies, removing it with the ovigera and transferring it to the mouth via the chelifores. *Achelia echinata*, *Endeis laevis* and *Pycnogonum litorale* were found feeding on detritus that had accumulated on various substrates such as bryozoan colonies. In the case of *Endeis*, the detritus was first broken down with the spines surrounding the mouth. Similar observations were reported on *Ammothella longipes* and *Endeis spinosa* by Soler-Membrives et al. [17], who found the latter species to be exclusively detritivorous. This might explain the loss of chelifores in that genus as opposed to the related Phoxichilidiidae, which have well-developed chelifores. Richards [46] reported *Nymphon orcadense* feeding on detritus of unidentified animal origin. Therefore, while specialized detritivory seems to occur only in *Endeis*, many pycnogonids appear to be able to feed on detritus when no other food is available.

### Other prey

Richards & Fry [12] suggested that pycnogonids might feed by filtering particle-rich water, suggesting that *Nymphon orcadense* uses this behavior when its preferred polychaete prey is not available. They noted that during these times the pycnogonid was observed to feed on other prey, but much less frequently than would be expected. Such a mode of feeding would also explain the observation that *Colossendeis proboscidea* was seen rapidly opening and closing its proboscis lips in “goldfish fashion” [12]. They also suggested that pycnogonids may be able to take up nutrients through the cuticle, which however has, to our knowledge, not yet been demonstrated.

Based on stable isotope analyses, Bergquist et al. [108] inferred that the hydrothermal vent species *Sericosura verenae* is mostly bacterivorous, while other *Sericosura* species may combine bacterivory with detritivory. Based on the same method, Cordes et al. [109] also inferred bacterivory in *Anoplodactylus* sp. from cold seeps.

Animal taxa other than those discussed in the previous section were also found to be pycnogonid prey. Zenker [19] found benthic foraminiferans in the proboscis of *Nymphon gracile*, which were probably ingested by consuming detritus. Shabica [96] recorded *Pentanymphon antarcticum* feeding on a small ctenophore. Richards [46] observed *Nymphon orcadense* feeding on the nemertean *Antarctonemertes valida*. Shabica [96] found *Colossendeis* sp. feeding on the nemertean *Parbolasia corrugatus* in the Antarctic. Soler-Membrives et al. [17] recorded two occurrences of predation by *Ammothella longipes* on unidentified nematodes. King & Crapp [110] found *N. gracile* feeding on eggs of the gastropod *Nucella*. Kott [111] found a specimen of *Ammothea carolinensis* whose proboscis was inserted into the branchial cavity of an ascidian (*Pyura georgiana*), apparently to feed on its genital products after release from the gonads. Lebrato & Jones [112] observed *Colossendeis* sp. feeding on pyrosome carcasses (*Pyrosoma atlanticum*). Leigh-Sharpe [113] recorded a specimen of *Pycnogonum litorale* found on the gills of a fish (*Merlangius merlangus*). Arnaud [114] and Arnaud & Bamber [2] recorded eight Antarctic pycnogonid species (*Nymphon australe*, *Pentanymphon antarcticum*, *Ammothea carolinensis*, *A. clausi*, *A. glacialis*, *Colossendeis megalonyx*, *C. robusta*, *C. scotti*) feeding on seal meat in fish traps. In an aquarium setting, *Nymphon orcadense* fed on a mixture of minced limpet, squid and spratt [46]. Richards [46] also observed that *Colossendeis* and/or *Decolopoda* apparently fed on smaller pycnogonids (*Nymphon orcadense*) in an aquarium setting. These observations demonstrate that many pycnogonids are generalist feeders, which are able to use a wide variety of food sources on which they are not specialized.

## General findings

Our review documented observations of feeding for only approximately 100 of the about 1500 species (Table 1, Additional file 1). Thus, the most important finding is that for most pycnogonid species, the feeding mode and preferred food still remains unknown. This is especially true of deep-sea forms as well as those of the Antarctic, which include about 20% of the known pycnogonid species [115]. Therefore, taxonomic groups which are typical of these regions, such as the Colossendeidae and Pallenopsidae, are also underrepresented here.

However, for those species where details about feeding items are known, the data reviewed here confirm the generally accepted view that pycnogonids feed mostly on sessile organisms such as hydroids, actinians and bryozoans. King [1] stated that littoral pycnogonids feed on hydroids, bryozoans and sponges “in about that order of frequency”. The data reviewed here show that hydroids are indeed the most common food source, being eaten by members of almost all pycnogonid families. It is also confirmed that the second most common food source is bryozoans, which are also consumed by a wide variety of pycnogonid species. However, there are only very few records of littoral pycnogonids feeding on sponges (e.g. [21]), which suggests that they are not among the preferred prey. Sponges might be a more common food source for deep-sea forms [1], although, so far, the data are insufficient. Other types of prey are used less commonly, often by specialist feeders (e.g. Pycnogonidae as actinian specialists). Sediment feeding appears to be especially common in deep-sea forms, about whose behavior little is known, and may be an important but underestimated part of pycnogonid feeding ecology, as already suggested by King [1]. Food sources of juvenile and adult pycnogonids should be distinguished, as there are several species (mostly ammotheids and ascorhynchids) which are parasitic even as late-stage juveniles but free-living as adults, such as the bivalve parasite *Nymphonella tapetis*.

### Food specialization as a rule?

Many pycnogonids appear to be specialized for feeding on a single taxonomic group such as thecate or athecate hydroids, actinians, or bryozoans (Additional file 1). Individuals of these species may even be unable to survive the absence of their preferred food [16]. Like other specialized feeders, these pycnogonids may be vulnerable to environmental change if the frequency of their prey item is reduced.

However, the claim [1] that no pycnogonids are dependent on a single host species (rather than a larger taxonomic group) appears to be correct. Hydroid feeders seem to be the most common group in temperate shallow seas, and feeding on hydroids is therefore particularly intensively studied. The feeding mechanisms of actinian specialists (Pycnogonidae) and detritivores (some *Endeis* species) have also been well studied. Other pycnogonids, especially members of the Phoxichilidiidae such as *Phoxichilidium* and *Anoplodactylus*, appear to be generalist feeders able to live on a wide variety of prey. Prell [39] already noted that *Phoxichilidium femoratum* is a voracious predator (“ein arges Raubtier”) of many different animals, and the observations of Lotz [16] and others on *Anoplodactylus* agree with this. It is notable that, even within a genus, the feeding preferences may vary widely. Examples are *Anoplodactylus*, which contains generalists as well as obligatory echinoderm commensals, *Endeis*, which includes detritivores and coral feeders, and *Austrodecus*, which includes bryozoan and hydroid feeders. Helfer & Schlottke [116] stated that pycnogonids, due to being incapable of making fast movements, are only able to feed on slow-moving or sessile prey. While this appears to be generally true, there are exceptions. Several pycnogonid species were observed to capture and eat errant polychaetes, and *Anoplodactylus* also consumes free-swimming crustaceans (see above).

Differences in feeding preference often correspond to differences in morphology. There are variations, especially in the morphology of the proboscis and chelifores, which can be assumed to correlate with feeding preferences, such as extremely thin proboscides in bryozoan-feeding austrodecids and *Stylopallene*, or the very robust chelifores of *Pseudopallene* and related genera used to crush bryozoan zooids. In *Anoplodactylus*, the lips appear to be specialized for cutting tissue, which would be useful for its generalist predatory lifestyle. The chelifores are well developed in most hydroid feeders, which use them to grasp stems or hydrothecae and lead them to the mouth. In animals living parasitically on much larger hosts, such as Pycnogonidae on actinians, and in detritivores such as *Endeis* they are reduced. The proboscis is much more mobile in ammotheids and ascorhynchids than in most other forms, which fits with the fact that these animals seem to be mostly hydroid feeders that cannot hold their prey in their small chelifores. However, these correlations may not be perfect. As an example, a preference for bryozoans has been shown for only one Antarctic austrodecid species [26], while the Australian *Austrodecus frigorifugum* feeds on hydroids [27]. For most taxa, the correlation between morphology and feeding has yet to be investigated. This is especially true of internal anatomy, where Wagner et al. [8] have found significant differences between taxa in the proboscis, and the detailed anatomy of the digestive system has been studied only for very few taxa.

### Cryptic species and food specialization

Morphological correlates of different feeding habits thus exist, but the question arises what those differences actually are. In other words, there is a considerable gap in the current knowledge of pycnogonid feeding ecology that needs to be closed. This is of particular interest since in the past decade molecular and morphological studies, especially in the Southern Ocean, have shown that pycnogonids are a useful model taxon for analyses of speciation and phylogeography of holobenthic marine organisms [3, 4]. However, these studies have focused exclusively on genetic drift as speciation motor, while selection (with food preferences as a major cue) has hardly been considered so far. Besides, differences in food preference between closely related species are little known. To deepen knowledge of pycnogonid feeding ecology would, therefore, be an important contribution to marine evolutionary biology, especially of high-latitude environments.

### New methods provide new insights

While most observations and experiments were conducted using classical setups, mostly by direct observation of feeding, only a few studies have been undertaken using novel techniques such as fatty acid analyses [17, 101] or stable isotopes [108, 109, 117]. Molecular content analyses of pycnogonid gut content have to our knowledge never been published. A metabarcoding approach, in which standard barcoding markers are amplified from bulk samples and sequenced with next-generation methods, has been successfully used for identifying gut contents in several taxa (e.g. [118]), and could also be useful in pycnogonids. However, as the cellular material is already processed and filtered in the pycnogonid proboscis, it does not enter the midgut [6]. Therefore, a metabarcoding approach might be less successful than in animals where cellular prey tissue is found in the gut. When genomic or transcriptomic data of pycnogonids become available (several transcriptomes already exist in unpublished form), they should be checked carefully for the presence of non-pycnogonid DNA, which could be an important source of new data on pycnogonid feeding. Preliminary results by J. Dömel and T. Macher (in prep.) for two Antarctic pycnogonid species have already confirmed the presence of several taxonomic groups known to be pycnogonid prey.

## Outlook

Despite the fact that pycnogonids have been observed for almost two centuries, information about the feeding habits of more than 90% of the species is missing. Hence, one of the tasks for future studies will be to keep going the “naturalist path”, i.e. observation of pycnogonids in their habitats in order to record their actual food preferences. Moreover, previous analyses of morphological adaptations of the organs of food assimilation (chelifores, palps, proboscis lips, proboscis inner structures) to the type of nourishment proved fruitful and therefore should be made for many more species. Apart from analyses of these structure-function relationships, there are three approaches using modern techniques that have been neglected until now, i.e. fatty acid and stable isotope analyses as well as DNA sequencing of gut contents. However, these results are needed to analyse the relative contribution of selection for pycnogonid speciation processes next to the typically discussed allopatric scenarios fuelled by genetic drift and lineage sorting.

## Conclusions

1. Pycnogonids feed on a wide variety of prey, mostly on sessile animals, but also detritus and other food sources.
2. Hydroids appear to be the most common food source of pycnogonids, followed by bryozoans and actinians. Other food sources are less common.
3. Many pycnogonids are generalist feeders, but a number of taxa are specialized in a particular food source, e.g. actinians for members of the Pycnogonidae.
4. Pycnogonid taxa often show clear adaptations to their preferred food, especially in the morphology of the proboscis and chelifores.
5. For most pycnogonids, especially deep-sea forms, the preferred food source is still unknown. More research on pycnogonid feeding ecology could reveal mechanisms of differentiation between closely related species and therefore of evolutionary radiations.

## Box 1 First reports were often erroneous

The first records of pycnogonid feeding were erroneous. To our best knowledge, Linnaeus [120] was the first who mentioned a pycnogonid, identified as *Phoxichilidium femoratum* by Calman [121], feeding by drilling holes with its proboscis into the shells of mussels (*Mytilus* spp.). However, this way of feeding appears to be physically impossible, as the tissue of the proboscis lips is certainly not hard enough to drill into a molluscan shell. Similarly erroneous was the claim by Lamarck [122] and others that *Pycnogonum* is parasitic on whales, which was based on confusion with cyamid amphipods [123]. The first reliable observations on pycnogonid feeding were given by Zenker [19], who reported on food being found in the dissected proboscis of *Nymphon gracile*. Parasitism on hydroids by pycnogonid larvae was first documented by Allman [124]. Adult pycnogonids have often been found on hydroids and other sessile organisms (e.g. [7]), but the first documented observation of feeding was published by Cole [36] for *Anoplodactylus lentus*. Further detailed observations on the feeding mode of several pycnogonid species belonging to different families were recorded by Prell [39]. Later, some authors also performed experiments in which the food preference of different pycnogonid species, mostly from the North Sea [16, 25], but also from the Southern Ocean [26] was tested.

A synopsis of pycnogonid-host associations was given by Helfer & Schlottke [116], however, not in all cases the pycnogonid can be assumed to be feeding on the organism on which it was found (Fig. 4 shows some associations of pycnogonids with other organisms, and it is unclear whether any of these are used as a food source). Some entries in their table are also erroneous, with the original sources actually describing epibionts or predation on pycnogonids. King [1] updated this synopsis, distinguishing between associations of larval and adult pycnogonids with their hosts and cases where the pycnogonids were actually observed feeding. A further short review of pycnogonid feeding was provided by Arnaud & Bamber [2] as part of their general review of pycnogonid biology.

## Additional file

Additional file 1: Feeding-table. Summary of known food sources for pycnogonid species. Reference numbers are the same as in the main text. (XLSX 50 kb)
